# Multilayer redox-based HfO_x_/Al_2_O_3_/TiO_2_ memristive structures for neuromorphic computing

**DOI:** 10.1038/s41598-022-22907-5

**Published:** 2022-10-29

**Authors:** Seongae Park, Benjamin Spetzler, Tzvetan Ivanov, Martin Ziegler

**Affiliations:** Micro- and Nanoelectronic Systems, Institute of Micro and Nanotechnologies MacroNano, Technische Universität Ilmenau, Ilmenau, Germany

**Keywords:** Nanoscale devices, Electronic devices, Electronic properties and materials

## Abstract

Redox-based memristive devices have shown great potential for application in neuromorphic computing systems. However, the demands on the device characteristics depend on the implemented computational scheme and unifying the desired properties in one stable device is still challenging. Understanding how and to what extend the device characteristics can be tuned and stabilized is crucial for developing application specific designs. Here, we present memristive devices with a functional trilayer of HfO_x_/Al_2_O_3_/TiO_2_ tailored by the stoichiometry of HfO_x_ (*x* = 1.8, 2) and the operating conditions. The device properties are experimentally analyzed, and a physics-based device model is developed to provide a microscopic interpretation and explain the role of the Al_2_O_3_ layer for a stable performance. Our results demonstrate that the resistive switching mechanism can be tuned from area type to filament type in the same device, which is well explained by the model: the Al_2_O_3_ layer stabilizes the area-type switching mechanism by controlling the formation of oxygen vacancies at the Al_2_O_3_/HfO_x_ interface with an estimated formation energy of ≈ 1.65 ± 0.05 eV. Such stabilized area-type devices combine multi-level analog switching, linear resistance change, and long retention times (≈ 10^7^–10^8^ s) without external current compliance and initial electroforming cycles. This combination is a significant improvement compared to previous bilayer devices and makes the devices potentially interesting for future integration into memristive circuits for neuromorphic applications.

## Introduction

Memristive devices are typically two-terminal resistive elements with a hysteretic current–voltage characteristic, which enables switching between different resistance states^1–3^. As analog resistive switching memory, memristive devices have emerged as one of the critical technologies for implementing neuromorphic computing systems^4,5^, i.e., systems that mimic the structure or working mechanisms of the brain^6^. For such computing systems, memristive devices offer a variety of potential advantages compared to traditional memory devices such as flash or SRAM^4^. The simple two-terminal device structure of memristive elements permits a high integration density in crossbar arrays^7,8^, and thereby, highly energy-efficient and parallelized in-memory computing. Additionally, the possibility of in-memory computing results in a low latency because it is not restricted by the data exchange between the memory and a central processing unit (von Neumann bottleneck)^4,5^.

A major challenge for memristive devices in neuromorphic systems is a controllable and reproducible setting of resistance states for storing synaptic weights in artificial neuronal networks (ANNs). This requires high endurance and reproducibility of the device properties over many switching cycles^4,9^. In general, the requirements of the device parameters depend on the kind of weight update of the respective neuromorphic system. For example, the spike-timing dependent plasticity (STDP) rule is mainly relevant for spiking neural networks (SNN) and requires a time dependent and nonlinear resistance change^10^. In contrast, classical ANNs (such as deep neural networks or convolutional neural networks) do not require time dependency, but instead a linear resistance change and as many stable resistance values as possible^11^. Although the requirements imposed on the memory duration are lower in many areas of neuromorphic computing than in nonvolatile memories^12,13^, too short state retention times preclude the devices from broad application in neuromorphic computing.

The properties of memristive devices depend on the underlying mechanisms of the resistive switching process, which can be broadly classified into filament-type switching and area-type switching^14,15^. Filamentary switching is characterized by the presence of (at least) one conductive filament, which provides an electrical pathway through the memristive material in the low resistive state. It typically must be induced with an initial forming procedure before operating the device^3,5^. The growth and disruption of the filament are stochastic processes and cause an abrupt change in the resistance between a high resistive state (HRS) and a low resistive state (LRS), usually without accessible intermediate resistance states (digital switching). Due to the high nonlinearity of the switching kinetics, additional electronics is typically required to stabilize the current–voltage characteristic by limiting the current through the device (current compliance). Limiting the current prevents uncontrolled filament formation and growth, leading to irreversible changes in the device characteristics and rendering the device useless^16^. From an application point of view, the highly nonlinear switching kinetics have enabled filamentary devices capable of long retention times > 10 years^17,18^, high switching speeds < 1 ns^19,20^, high endurance > ${10}^{12}$ cycles^7,21^, and large on–off ratios (> 1000)^5,22^. These properties have made them attractive as a potential technology for nonvolatile memory approaches^23^. Major challenges are the low reproducibility of device parameters and endurance deterioration because of the underlying stochastic process^22^, but also the required initial forming step and additional electronics for the current compliance, which can be disadvantageous for the integration of the devices into large arrays^4^. On the other hand, area-type switching is a less mature technology^14^. Depending on the specific device, various potentially involved microscopic processes are under debate^24,25^. Generally, the current through the device is assumed to be dominated by interface-limited conduction processes, such as the tunneling or emission through/over interface potential barriers. Switching has been explained as a modulation of the barrier via the drift and accumulation of mobile charge carriers under the application of an external voltage. In this context, electromigration of mobile vacancies has been discussed^26–30^, as also the trapping dynamics of electrons^31,32^ and the uniform modification of bulk conduction mechanisms^14^. Because of the spatially uniform contribution of conduction mechanisms to the total current, the conductance scales with the device area and the stochastic components of the microscopic processes average and yield more reproducible macroscopic device properties than filamentary devices. Area-type devices can be fabricated with a low variation in the device characteristics and a correspondingly high yield^33^. Additional advantages are many intermediate and continuously accessible resistance states (analog switching) and often the possibility of operating without initial forming cycles or external current compliance^34–36^. However, the main challenges of area-type devices are a typically short retention time (seconds to days) and large switching times (> 1 ms) compared to filamentary devices^12,37^. A combination of the beneficial properties of both switching types is generally desirable.

Among various types of memristive devices investigated for neuromorphic computing^1,5,9,38–40^, memristive bilayer and multilayer oxide structures based on hafnium oxide and titanium oxide gained attention for the application as artificial synapses^35,41–43^. Such oxide structures can show filamentary- or area-type switching^23,42^, depending on the details of the respective layer system. Compared to hafnium oxide single-layer devices, HfO_x_/TiO_x_ bilayer structures demonstrated a variety of improvements^43^, such as a smaller variability^44^ and improved resistance modulation linearity^45^, and a larger number of resistance states^46^. These bilayer structures are mainly of the filamentary type and often still require current compliance and an initial electroforming step. Previous studies^47,48^ suggest that the filament formation in such systems is connected with the concentration of oxygen vacancies in the HfO_x_ layer and oxygen ions in the TiO_x_ layer. According to this work, the ions and vacancies are injected preferably at the HfO_x_/TiO_x_ interface by a voltage-induced creation of Frenkel pairs, owing to much higher activation energy in the bulk material^47,48^. Consequently, altering the layer structure and interfaces could represent a way of modifying the switching type and further improve the device characteristics for the applications pursued.

This work presents memristive trilayer devices with a functional layer stack of HfO_x_/Al_2_O_3_/TiO_2_ sandwiched between TiN and Au electrodes. We discuss the influence of the additional aluminum oxide layer, operating conditions, and the composition of the hafnium oxide layer on the resistive switching characteristics. A model is devised to provide a microscopic interpretation of the experimental results and derive further implications for the design of memristive multilayer elements. The paper is structured as follows: after presenting the details of the device materials and geometries in “Memristive devices and materials”, we analyze the current–voltage characteristics under different voltage operating conditions and for two different hafnium oxide compositions in the section “Current–voltage characteristics”. In “Discussion of the switching mechanisms”, the indications found for the underlying switching mechanisms are interpreted before they are quantified with a physics-based device model presented in “Physics-based compact model”. Afterwards, we discuss application relevant device characteristics in “Characterization for neuromorphic applications”, namely the state retention time and the linearity in the conductance update. Finally, the results are summarized, and a conclusion is drawn.

## Results

### Memristive devices and materials

**Figure 1:**
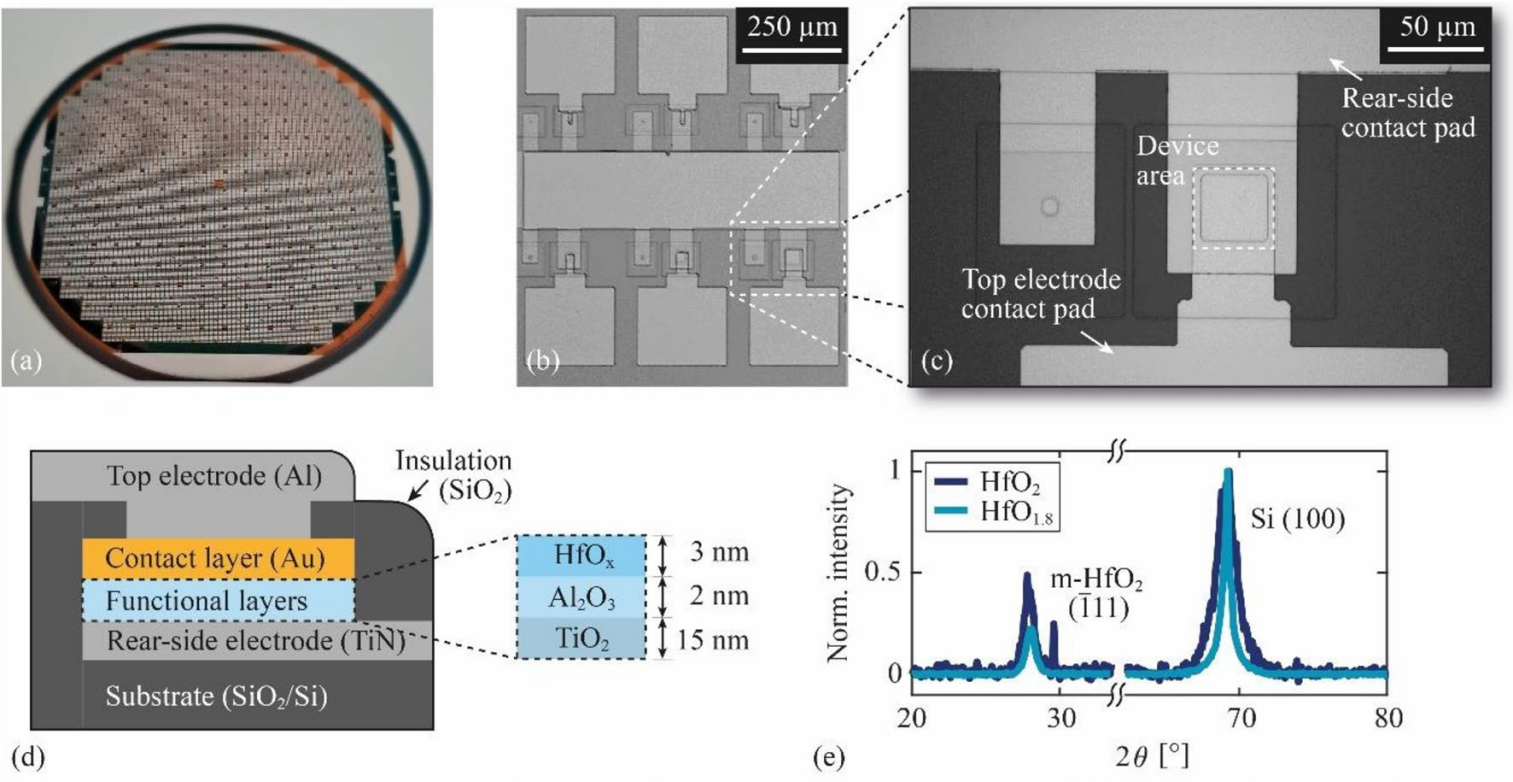
Illustration of the fabricated devices (**a**) 4-in. wafer with around 40,000 memristive devices, (**b**) arranged in groups of six with different device areas, (**c**) close-up top view of an individual device showing the top and rear-side electrode contact pads, and the device area is indicated. (**d**) Cross-sectional sketch along the dashed line in (**c**) of an example device with the configuration of the functional layers. (**e**) Normalized and background subtracted XRD intensity spectra of hafnium oxide, deposited with a dynamic (HfO_2_) and a static (HfO_1.8_) sputtering method on a Si substrate, indicating the presence of nanocrystalline m-HfO_2_.

All memristive devices presented here are fabricated with a 4-in. wafer technology, providing around 40,000 individual devices per wafer (Fig. 1a), arranged in groups of six (Fig. 1b). Each device can be electrically contacted via individual contact pads for the top electrode and a shared contact pad for the rear-side electrode. The top and rear-side electrodes are electrically insulated by a 180-nm-thick SiO_2_ layer, encapsulating the functional layers underneath. The top electrode contacts a 30-nm-thick Au layer, located under the SiO_2_ and on the top of the functional layer stack. Because the Au layer translates the electric potential applied to the top electrode to the functional layers, the lateral dimensions of the Au layer are used to define a device area $A$, as indicated by the dashed square in Fig. 1c. Within each group of six devices, the device area varies from a minimum of ${10}^{2}$ µm^2^ to a maximum of ${50}^{2}$ µm^2^. A schematic cross-section of the entire layer stack is illustrated in Fig. 1d. The functional layer stack comprises a sequence of HfO_x_(3 nm)/Al_2_O_3_(2 nm)/TiO_2_(15 nm), sandwiched between the Au contact layer and the TiN rear-side electrode. Here, the HfO_x_ is intended to introduce memristive behavior, the Al_2_O_3_ to modify the interface properties and the TiO_2_ layer is beneficial because it forms well-defined interfaces with the TiN electrode and the Al_2_O_3_ interlayer. While the thickness of the layers is kept constant throughout the various devices analyzed, we focus on two stoichiometries of the hafnium oxide (HfO_x_) layer with $x\approx 1.8$ and $x\approx 2$. The stoichiometries were confirmed by x-ray photoelectron spectroscopy (XPS) measurements on a test wafer with estimated uncertainties of ± 1%.

The structural properties are determined with x-ray diffraction (XRD) measurements on hafnium oxide layers deposited on 4-in. Si wafers. The normalized and background subtracted intensity spectra in Fig. 1e show two distinct peaks at $2\theta =69^\circ$ and $2\theta =28^\circ$. While the former corresponds to the (100) planes of the Si substrate, the latter can be assigned to the $\left(\overline{1 }11\right)$ planes of monoclinic HfO_2_ (m-HfO_2_)^49^. Therefore, we assume the presence of nanocrystalline m-HfO_2_ in our films, as demonstrated in previous investigations on hafnium oxide thin films^50–52^. From the difference in the intensity of the m-HfO_2_ peak, larger average grain size and better oriented $\left(\overline{1 }11\right)$ planes are expected in the HfO_2_ sample compared to the HfO_1.8_ sample^50^.

### Current–voltage characteristics

**Figure 2:**
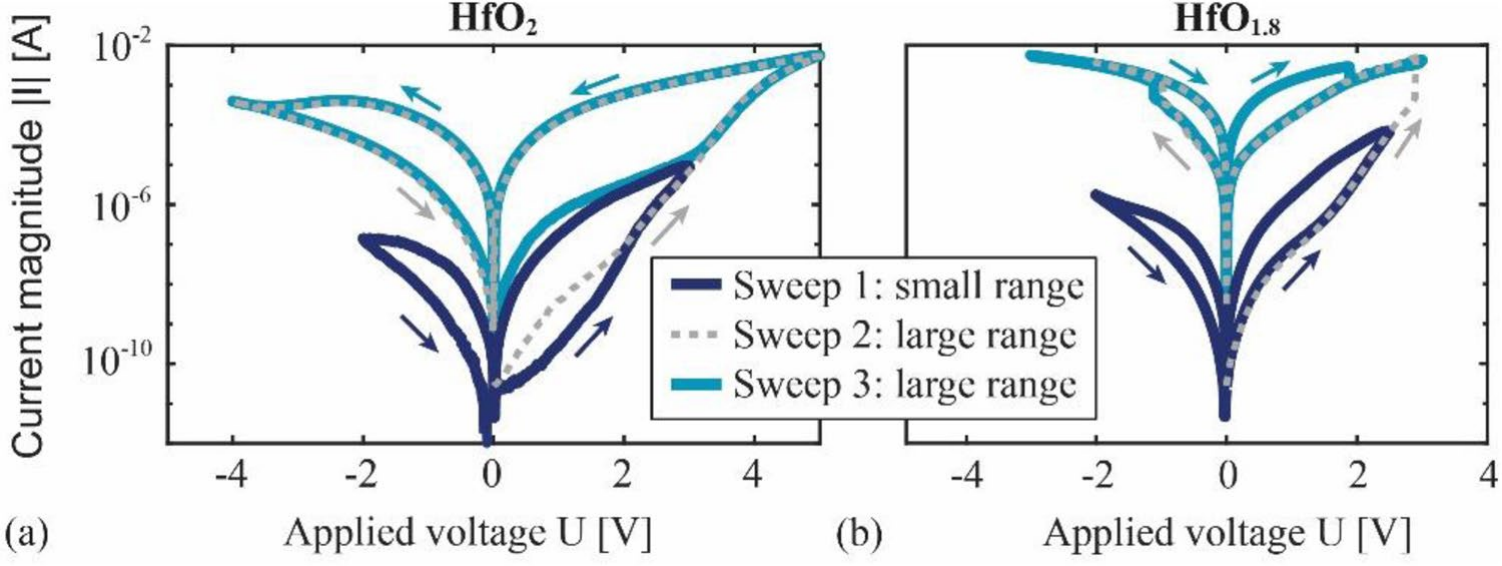
Results of three subsequently performed current–voltage measurements on two representative memristive devices with different stoichiometries of the HfO_x_ layer. (**a**) Measurements on a device with an HfO_2_ layer and (**b**) device with an HfO_1.8_ layer. The voltage was swept piecewise linearly, starting and ending at *U* = 0 V for 45 s.

We conducted quasi-static measurements of the current–voltage (I-U) hysteresis on two representative example devices with a device area of $A={35}^{2}$ μm^2^, to identify the influence of the stoichiometry of the HfO_x_ layer on the electric device characteristics. All measurements were performed at room temperature without current compliance, within a time of *t* = 45 s per loop. The obtained I-U curves are shown in Fig. 2. Additional measurements on various devices demonstrate stable hysteresis curves over at least 100 cycles with only minor deviations in the current magnitudes (“Methods”).

After an initial voltage sweep (Sweep 1 in Fig. 2), two curves (Sweep 2, Sweep 3 in Fig. 2) with an increased range of the applied voltage $U$ were measured. The results of the three voltage sweeps are presented in Fig. 2a (HfO_2_) and Fig. 2b (HfO_1.8_). In the initial voltage sweep (Sweep 1), the I-U curves of both samples show a similarly distinct hysteresis with a smooth transition between the high resistive state (HRS) and the low resistive state (LRS). The I-U characteristics change notably upon increasing the voltage range in the second measurement (Sweep 2). The larger voltage range in both samples reveals a transition to a second hysteresis curve with a significantly larger maximum current and lower resistance. While this transition occurs continuously in the HfO_2_ device, a jump is visible in the I-U curve of the HfO_1.8_ device. An additional measurement (Sweep 3) confirms the reproducibility and irreversibility of the low-resistance hysteresis curves and reveals further differences and features in the I-U characteristics of the two samples. In the HfO_2_ device, the hysteresis area increases compared to the initial sweep (Sweep 1), and the curve remains smooth. In contrast, in the HfO_1.8_ device, the hysteresis area reduces, and the direction of the hysteresis loop is reversed. The transition from the HRS to the LRS occurs via discontinuities at *U* ≈ − 1 V and back at *U* ≈ 2 V. While such discontinuities are an indication of filament-type switching^53^, the continuous resistance change observed in all small-range sweeps and the large-range sweep of the HfO_2_ device, is a typical characteristic of area-type switching^24^.

**Figure 3:**
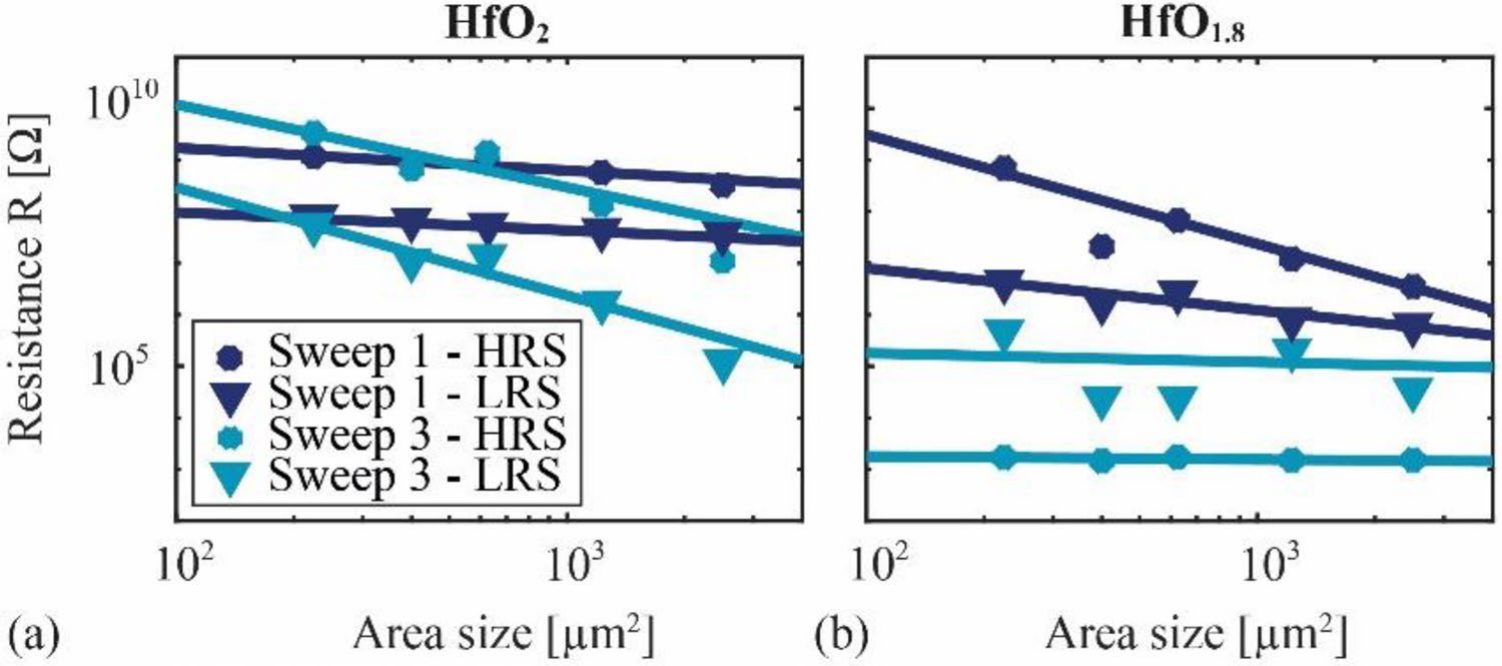
Dependency of the device resistances *R*_HRS_ and *R*_LRS_ on the active device area $A$ in the high resistive state (HRS) and low resistive state (LRS), respectively, and for both voltage ranges. The plotted mean values were obtained from measurements on many devices, with (**a**) HfO_2_ layers and (**b**) HfO_1.8_ layers.

These indications for the type of switching mechanisms can be confirmed by analyzing the dependency of the resistance $R:=U/I$ on the device area. For that, we measured the I-U curves of many samples with different device areas $A$ in both voltage ranges to extract the resistances in the high and the low resistive state, labeled as *R*_HRS_ and *R*_LRS_, respectively. The mean values of *R*_HRS_ and *R*_LRS_ are plotted in Fig. 3a (HfO_2_), and Fig. 3b (HfO_1.8_), together with fits of the data sets to guide the eye. The relative standard deviations of the mean values are provided in Table 2 (“Methods”) because they are so small that they would not be clearly visible in Fig. 3, which indicates a good device-to-device reproducibility. A reduction of *R*_HRS_ and *R*_LRS_ with increasing device area is visible in all measurements of the small voltage range (Sweep 1). This trend is stronger in the measurements of the large voltage range (Sweep 3) of the HfO_2_ devices, while no clear dependency on the area is visible in the large voltage range measurements on the HfO_1.8_ devices (Sweep 3, HRS). Consistent with the I-U measurements, the area independent resistance of the HfO_1.8_ devices supports the presence of a dominant filamentary conduction mechanism, and the area dependency of all other configurations confirms a dominant area-type conduction mechanism^14,23^. Hence, we identified two different switching mechanisms in the same device, as well as their dependency on the voltage treatment and the composition of the hafnium oxide layer. An explanation for the observed behavior, i.e., the reversed hysteresis direction, the voltage tunability of the I-U characteristics, and the current self-compliance, requires a discussion of the underlying switching mechanisms in the following section.

### Discussion of the switching mechanisms

The current–voltage characteristics and the observed change from an area to a filament-type switching behavior are explained in this section with a qualitative model considering the presence and dynamics of charged point defects. Based on the assumptions and measurements, we then estimate the formation energy of these defects.

**Figure 4:**
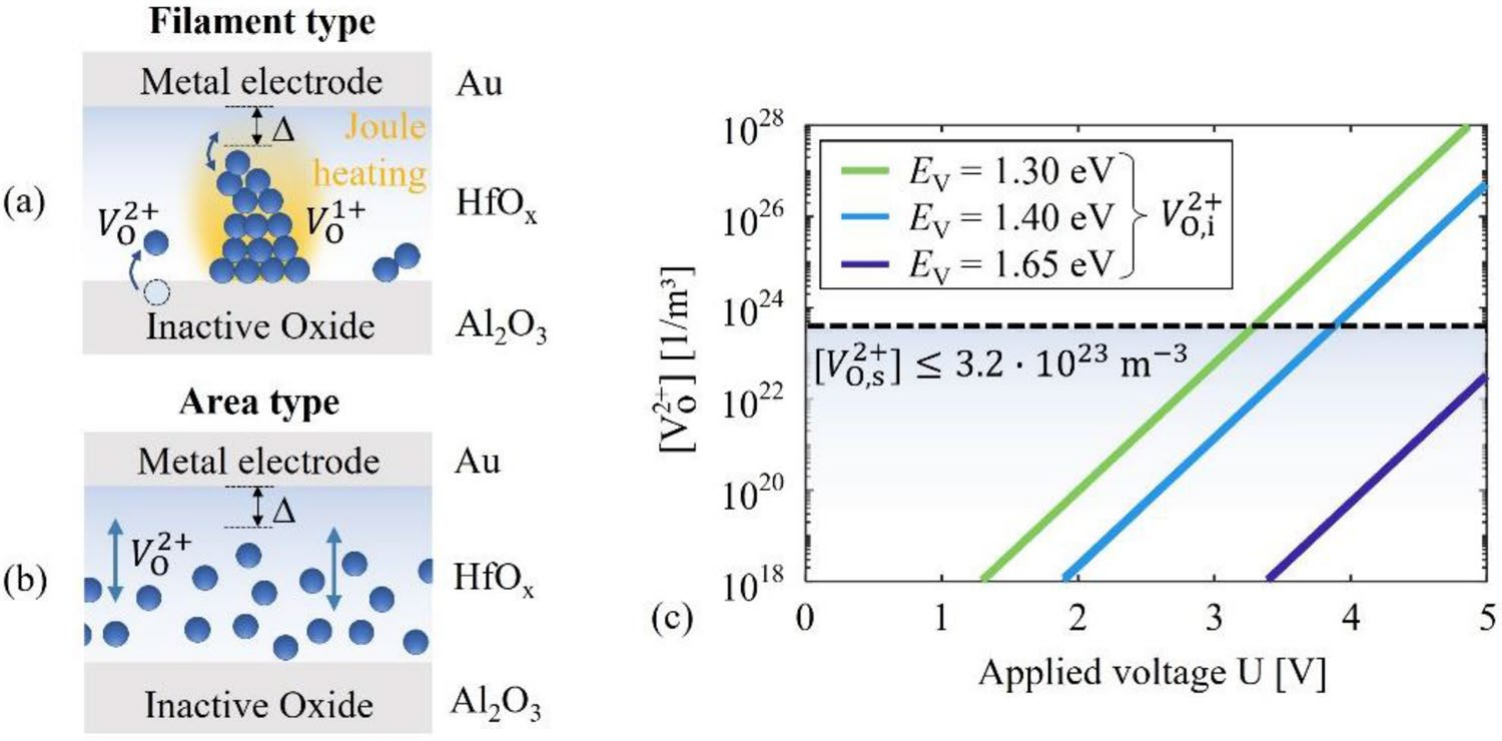
Illustration of the role of defect dynamics for the two switching types and the formation of oxygen vacancies ${V}_{\mathrm{O}}^{2+}$ at the interface. (**a**) Filamentary switching is based on the formation, growth, and disruption of an oxygen vacancy filament in defect rich HfO_x_. (**b**) Area-type switching in highly stoichiometric hafnium oxide, where the drift and diffusion of mobile vacancies change the potential landscape. (**c**) Density [${V}_{\mathrm{O}}^{2+}$] of double positively charged oxygen vacancies ${V}_{\mathrm{O}}^{2+}$ in the HfO_2_ layer, calculated as a function of the applied voltage *U* and plotted together with the stoichiometric vacancy density $\left[{V}_{\mathrm{O},\mathrm{s}}^{2+}\right]$ (maximum indicated by black, dashed line) in the HfO_2_ layer, which is still consistent with the XPS measurements. The electric field-induced vacancy density $[{V}_{\mathrm{O},\mathrm{i}}^{2+}]$ is plotted for three different formation energies *E*_v_.

Following ab initio calculations, double positively charged oxygen vacancies ${V}_{\mathrm{O}}^{2+}$ and negatively charged interstitials ${O}_{\mathrm{i}}^{2-}$ are mobile in HfO_2_ with activation energies of diffusion of 0.69 eV (${V}_{\mathrm{O}}^{2+}$)^54,55^ and 0.6 eV (${O}_{\mathrm{i}}^{-2}$)^56^. However, their formation via the Frenkel mechanism ${O}_{\mathrm{O}}^{0}\to {V}_{\mathrm{O}}^{2+}+{O}_{\mathrm{i}}^{2-}$ in the bulk of HfO_2_ can be omitted at room temperature owing to large activation energies of 5–9 eV^57,58^, also in the presence of high electric fields^58^. Instead, oxygen vacancies could be created at the HfO_x_/Al_2_O_3_ interface by incorporating oxygen into the aluminum oxide layer, functioning as an oxygen reservoir/scavenger, which increases the formation energy of oxygen interstitials and reduces the formation energy of oxygen vacancies^59^. This mechanism was shown to be energetically favored over the formation of Frenkel pairs in the bulk of hafnium/hafnium oxide layers^60^, and could similarly apply here. Oxygen vacancies are also expected to be introduced during the sputtering process with densities depending on the stoichiometries of the deposited films. Therefore, the switching processes in our devices are likely dominated by the dynamics and formation of ${V}_{\mathrm{O}}^{2+}$ as illustrated in Fig. 4, while oxygen interstitials can be omitted.

The stoichiometry of the HfO_1.8_ layer is close to the computationally estimated ideal stoichiometry (HfO_1.5_–HfO_1.75_)^61^ for the nucleation of a filament of oxygen vacancies (Fig. 4a). This process might be further fostered by the voltage-induced formation of vacancies during the large-range voltage sweep (Fig. 2b). Following the results of ab initio calculations^55,62^, the double-positive charge state of oxygen vacancies is only energetically favored over single-charged vacancies if the vacancies are sufficiently apart from each other. As ${V}_{\mathrm{O}}^{2+}$ vacancies reach a filamentary cluster, they preferably trap charges to occupy a neutral or single-charged state. The single-charged vacancies ${V}_{\mathrm{O}}^{+}$ are immobile in the bulk with activation energies of diffusion of $\approx 2\; \mathrm{eV}$^55^ but comparatively mobile within and alongside the filament with much smaller activation energies of 1.05 eV and 0.8 eV, respectively^55^. Vacancies in these charge states can return to the mobile double-charged state (cohesion-isolation transition) by releasing electrons. The transition energy barrier (≈ 1 eV) corresponds to electric field magnitudes, which can be achieved at small applied voltages of a few volts^55,62^. Further, the large current densities expected in the filament cause significant Joule heating and a corresponding increase in the defect mobilities^63^. Via these mechanisms, the filament can grow, and an applied voltage can modulate the electrode-filament gap and the interface potential. From our measurements, the filamentary switching process in the HfO_1.8_ device is also connected with a reversed direction of the hysteresis loop. This reversal could indicate a local change from n-type to p-type conduction^23^ because a reduced interface barrier for electrons corresponds to an increased barrier for holes. Consistent with experimental results, a change from n-type to p-type conduction can be induced in HfO_x_ by increasing the density of oxygen vacancies^64^.

Area-type resistive switching is assumed to occur at small vacancy densities, where filament formation is not favored, by the homogeneous drift and formation of ${V}_{\mathrm{O}}^{2+}$ as illustrated in Fig. 4b. Within this model, the charged point defects alter the interface potential as they drift in the applied electric field, which changes the current through the device. The transition of the measured I-U characteristics (Fig. 2) to a larger hysteresis with a smaller overall resistance can be explained by the voltage-induced formation of additional vacancies at the HfO_x_/AlO_x_ interface. The range of the required formation energy of this process can be estimated from the expected density of oxygen vacancies and the voltage range in which it is expected to increase significantly, i.e., the voltage range of the large-range voltage sweep in Fig. 2a. We use a rate equation to describe the formation of electric field-induced vacancies, derived from the Butler-Volmer equation^47^. Further, considering the uncertainty of ± 1% in the stoichiometry (“Memristive devices and materials” section) and the density of oxygen sites in ideal monoclinic HfO_2_, we estimate that the density $[{V}_{\mathrm{O},\mathrm{s}}^{2+}]$ of stoichiometric vacancies ${V}_{\mathrm{O},\mathrm{s}}^{2+}$ in our HfO_2_ layers must be $[{V}_{\mathrm{O},\mathrm{s}}^{2+}]\le 3.2\cdot {10}^{23} \;{\mathrm{m}}^{-3}$ to be consistent with the XPS measurements. Example results of the electric field-induced vacancy density $[{V}_{\mathrm{O},\mathrm{i}}^{2+}]$ as a function of the applied voltage $U$ are plotted in Fig. 4c. Results are shown for three different formation energies ${E}_{\mathrm{V}}$ together with the maximum of $[{V}_{\mathrm{O},\mathrm{s}}^{2+}]$ indicated by a black dashed line. From the current–voltage measurements in Fig. 2a, a significant increase in the vacancy density is expected for applied voltages between 3 and 5 V, which corresponds to a formation energy of approximately ${E}_{\mathrm{V}}>1.4 \;\mathrm{eV}$ in Fig. 4c if we assume that the maximum of $\left[{V}_{\mathrm{O},\mathrm{s}}^{2+}\right]$ is present in the HfO_2_ layer. For smaller formation energies $[{V}_{\mathrm{O}}^{2+}]$ reaches unrealistically high values at $U = 5 \mathrm{V}$, e.g., $\left[{V}_{\mathrm{O}}^{2+}\right]>{10}^{28}1/\mathrm{m}^{2}$ for ${E}_{\mathrm{V}}\approx 1.3 \; \mathrm{eV}$. While such high vacancy densities have been used for filamentary regions^65^, we consider it too high for area-type switching because it by far exceeds the density of oxygen lattice sites of $\approx 3.2\cdot {10}^{25}\;{\mathrm{m}}^{-3}$ in ideal monoclinic HfO_2_. A more precise estimation of ${E}_{\mathrm{V}}$, including an upper limit, requires knowing the range of the vacancy density in the HfO_2_ layer. For such an estimation, a model is presented in the next section.

### Physics-based compact model

**Figure 5:**
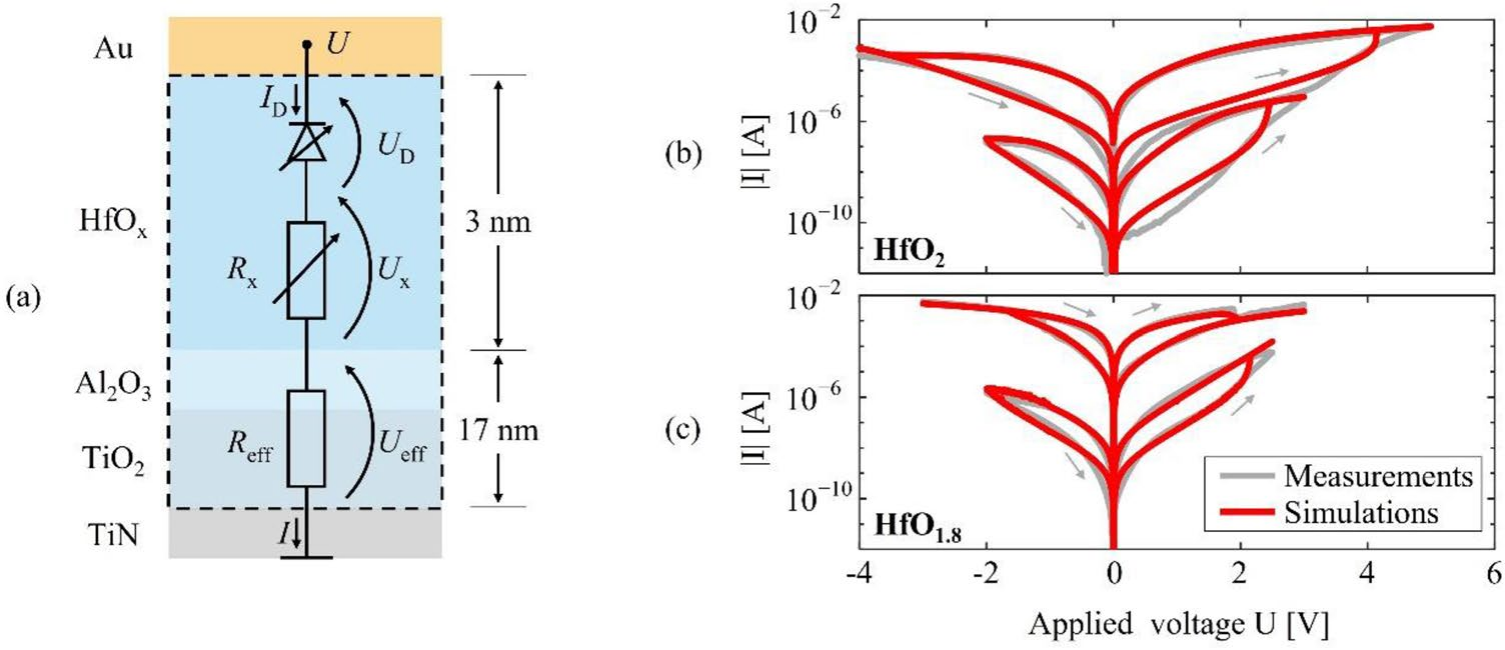
Comparison of simulations with measurements of the current–voltage characteristics. (**a**) Illustration of the equivalent circuit of the physics-based compact model used. (**b**) Comparison of simulations with measurements from Fig. 2 over the small-range and the large-range voltage sweep of the HfO_2_ device and (**c**) the HfO_1.8_ device.

The following model is based on a simplified version of the mechanisms described in Section C, and it will be used to discuss the physics of the switching processes quantitatively. The equivalent circuit of the model is shown in Fig. 5a.

Within the model, we assume that the electromigration of mobile double-charged oxygen vacancies ${V}_{\mathrm{O}}^{2+}$ in the hafnium oxide layer dominates the hysteresis in the current–voltage characteristics. Based on this assumption, we represent the contribution of the other layers by an effective resistance *R*_eff_ and omit mobile ions in the TiO_2_ layer. This is justified by its large thickness and correspondingly small electric field compared to the HfO_x_ layer. For area-type switching, the resistance *R*_x_ of the HfO_x_ layer is approximated to be constant depending only on the average dopant concentration, while for filamentary switching, it is a function of the filament length. The current through the Au/HfO_x_ interface is represented by a diode current *I*_D_ and follows a modified Shockley equation with a barrier potential and ideality factors that depend on the concentration of mobile dopants at the HfO_x_/Au interface. The dynamics of ${V}_{\mathrm{O}}^{2+}$ is modeled using the expression of Mott and Gurney^66^ for the ionic current as a function of the electric field. Capacitive effects are neglected in good approximation because of the low frequencies used in all measurements here. On this time scale, also Joule heating can be calculated using the stationary equilibrium solution and an effective thermal resistance, which unifies heat loss.

Despite the approximations made in the model, the simulations fit the measured current–voltage characteristics very well (Fig. 5b,c). Distinct features such as the discontinuities and the reversed direction of the hysteresis in the filament-type measurement are reproduced (Fig. 5c), as well as the nonlinearities and asymmetry of the maximum currents. With ≤ 0.1 eV, the values for the Schottky barrier lowering found in the area-type devices are of a typical order of magnitude reported previously^67^. Also, the obtained activation energies of diffusion (≈ 0.4–0.7 eV) roughly match the theoretical value of 0.69 eV^54,55^ for ideal single crystalline HfO_2_ but are overall smaller. Such a deviation is expected because of the reduced activation energy of diffusion along the grain boundaries present in our HfO_2_ layer. Minor deviations occur mainly around the maximum positive voltage in the simulations of the area-type curves and might be a consequence of the simplified description of the dopant dynamics and of omitting inhomogeneities. Further, additional contributions to the hysteresis from the dynamics of electrons and holes being trapped and released from interface or volume trap states cannot be entirely excluded^24^. Such trap states are introduced by the ${V}_{\mathrm{O}}^{1+}$ and ${V}_{\mathrm{O}}^{2+}$^68–76^, and therefore, their contribution to the hysteresis could change with the applied voltage, composition, and microstructure^76,77^. Electron trap states have been analyzed in other Al_2_O_3_/HfO_2_ structures, which were designed as charge-trapping memories with retention times of up to ${10}^{8}$ s^78–80^. Such a mechanism could contribute to the hysteresis curves in our devices, too.

From the model, we obtain a vacancy density range of approximately ${10}^{18}-{10}^{21} \;{\mathrm{m}}^{-3}$ for the low-defect area-type devices (HfO_2_) and consistently much larger values of $5\cdot {10}^{23}-6\cdot {10}^{24} \;{\mathrm{m}}^{-3}$ for the high-defect area-type device (HfO_1.8_). These values match the expected stoichiometric vacancy densities of $[{V}_{\mathrm{O},\mathrm{s}}^{2+}]<3.2\cdot {10}^{23} \;{\mathrm{m}}^{-3}$ (HfO_2_) and $\left[{V}_{\mathrm{O},\mathrm{s}}^{2+}\right]=3.2\cdot {10}^{24} \;{\mathrm{m}}^{-3}$ (HfO_1.8_), and are smaller than the vacancy densities obtained for the filament-type device ($\approx {10}^{25}-{10}^{27} \;{\mathrm{m}}^{-3}$) in good agreement with previous investigations^44,65^. Hence, the results reflect the described microscopic mechanisms very well. Using the range of the vacancy density in the HfO_2_ device, we estimate an energy of approximately ${E}_{\mathrm{V}}\approx 1.65 \;\mathrm{eV}\pm 0.05 \;\mathrm{ eV}$ as indicated in Fig. 4 for the formation of ${V}_{\mathrm{O}}^{2+}$ at the Al_2_O_3_/HfO_2_ interface. This value is much smaller than the formation energy 5–9 eV^57,58^ of Frenkel pairs in the bulk and larger than $0.9 \;\mathrm{eV}$^47^ for forming Frenkel pairs at TiO_x_/HfO_x_ interfaces of bilayer devices without stable area-type switching. The result is consistent with our assumptions and with the fact that Al_2_O_3_ is known as a poor oxygen vacancy former^62^ with large activation energy for the diffusion of oxygen ions^45,81^. Based on the above estimations, the aluminum oxide layer is expected to stabilize the area-type switching process by limiting the formation and dynamics of oxygen vacancies, which is also quantitatively consistent with our model.

### Characterization for neuromorphic applications

Two important device properties for neuromorphic applications are the time a device remains in the low resistive state after a set pulse was applied (state retention time) and the linearity of the conductance as a function of the number of applied SET and RESET pulses (linearity in conductance update). In the following, we characterize these two properties for the two different compositions and the two voltage ranges.

The retention characteristics of the LRS can be measured as the resistance ${R}_{\mathrm{on}}$ of the LRS as a function of time *t*. To obtain ${R}_{\mathrm{on}}\left(t\right)$, the respective memristive device is initially set to the LRS of either the large-range or the small-range voltage characteristic (Fig. 2) by applying a rectangular voltage pulse with a correspondingly large or small voltage amplitude. The evolution of ${R}_{\mathrm{on}}\left(t\right)$ is probed with a series of reading voltage pulses and normalized to the corresponding resistance ${R}_{\mathrm{off}}$ in the HRS. The resulting on–off ratio ${R}_{\mathrm{on}}\left(t\right)/{R}_{\mathrm{off}}$ is evaluated by fitting the Curie-von Schweidler power law ${R}_{\mathrm{on}}/{R}_{\mathrm{off}}=\beta {t}^{\alpha }$^82^ to the measurements with fitting parameters $\beta$ and $\alpha$. Besides describing the dynamics of capacitive discharge and charge trapping^82–85^, this power law has been used previously to characterize the retention characteristics of double-barrier memristive devices^34^ and is used here to enable a quantitative comparison. We define the state retention time ${t}_{\mathrm{r}}$ via ${R}_{\mathrm{on}}\left(t={t}_{\mathrm{r}}\right)/{R}_{\mathrm{off}}=1$ and estimate it by extrapolating the measured data with the power law.

**Figure 6:**
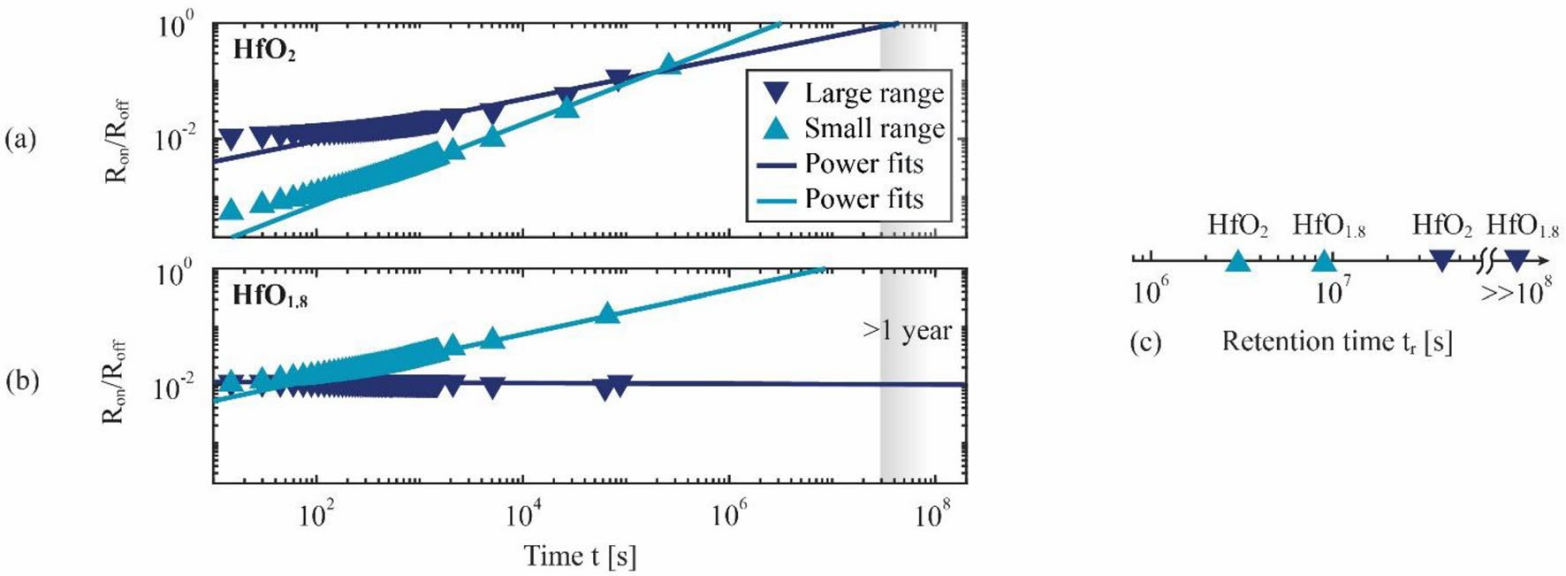
Measured resistance ratios ${R}_{\mathrm{on}}/{R}_{\mathrm{off}}$ as a function of time and power-law fits of the function ${R}_{\mathrm{on}}/{R}_{\mathrm{off}}=\beta {t}^{\alpha }$ for (**a**) the HfO_2_ device and (**b**) the HfO_1.8_ device. (**c**) Retention time ${t}_{\mathrm{r}}$ for the two hafnium oxide compositions and the two voltage ranges, estimated from the values of the power functions at ${R}_{\mathrm{on}}/{R}_{\mathrm{off}}=1$.

The results of the measurements and fits are presented in Fig. 6a (HfO_2_) and b (HfO_1.8_), and the calculated retention times ${t}_{\mathrm{r}}$ are summarized in Fig. 6c. Overall, the fits match very well, and the on–off ratios follow the expected power law. A clear time dependency is visible in all data sets except for the measurement on HfO_1.8_ with a large set voltage amplitude. Within the measurement accuracy ${R}_{\mathrm{on}}/{R}_{\mathrm{off}}$ is constant for this data set over the entire period tracked, which indicates a retention time ${t}_{\mathrm{r}}\gg {10}^{8}$ s. All other values of ${t}_{\mathrm{r}}$ are between some ${10}^{6}{-}{10}^{7}$ s. For both samples, a longer retention time ${t}_{\mathrm{r}}$ is observed in the measurement series with a large set-voltage amplitude compared to those with a small set-voltage amplitude. More specifically, the retention time can be improved by choosing the corresponding set voltage amplitude from $\approx 30$ days to $\approx 460$ days (HfO_2_) and from $\approx 115$ days to an estimate of several years (HfO_1.8_).

Such high retention times of several years are often observed in filament-type devices^17,18^, while much shorter retention times are reported for area-type devices^24,34^. Consequently, the results from the retention measurements are consistent with the analysis of I-U characteristics in Section B.

Another critical parameter for the application of memristive devices in neuromorphic computing is the linearity in the conductance as a function of the number of applied SET pulses. For linearity analysis, pulse measurements are conducted on the two memristive devices from Fig. 2, starting in the HRS of either the small-range or large-range voltage characteristics. A series of 21 SET voltage pulses and 20 RESET voltage pulses are applied, all rectangular and with a pulse length of 50 ms. A reading voltage pulse follows every SET and RESET pulse to probe the conductance.

**Figure 7:**
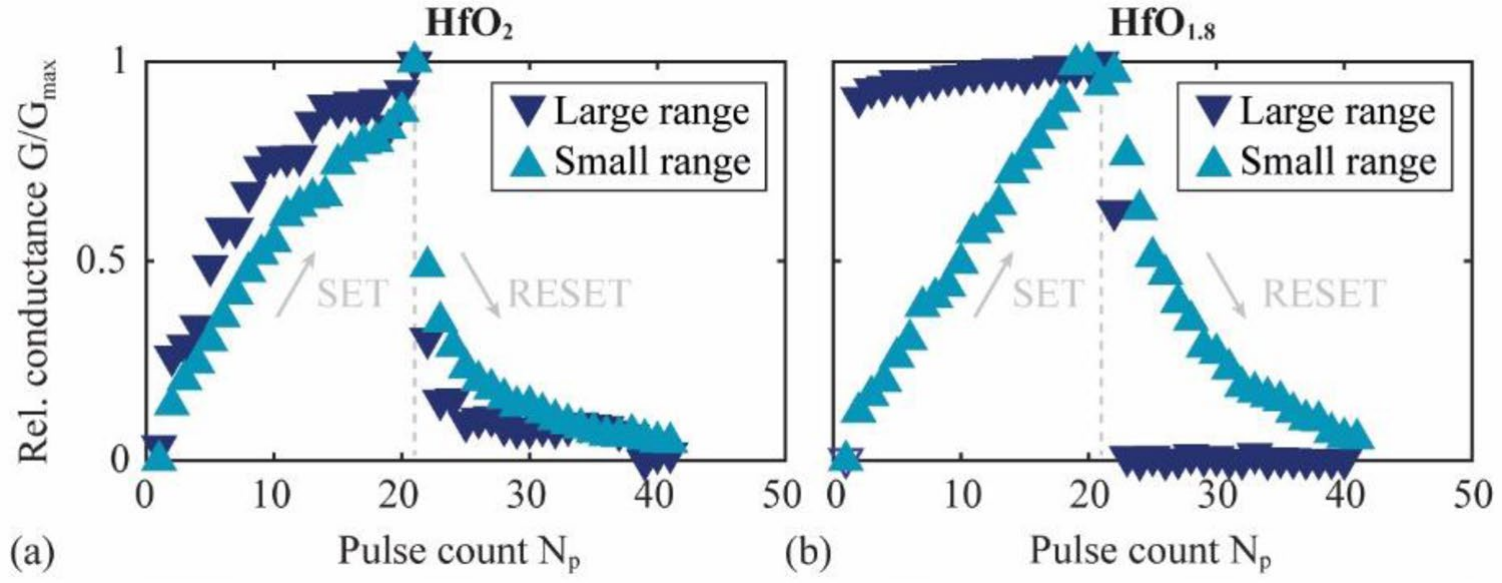
Conductance ${\text{G}}/{\text{G}}_{\text{max}}$ normalized to the maximum value ${\text{G}}_{\text{max}}$ of the conductance *G* as a function of the number of applied voltage pulses N_p_ for (**a**) the HfO_2_ device and (**b**) the HfO_1.8_ device, and two different amplitude ranges of the applied SET/RESET voltage pulses.

The measured conductance $G$ is normalized to its maximum value ${G}_{\mathrm{max}}$ and plotted as a function of the pulse count ${N}_{\mathrm{P}}$ in Fig. 7a (HfO_2_ device) and Fig. 7b (HfO_1.8_ device). In the measurements on both devices, a significant difference can be observed depending on the voltage range used for the SET pulses. For the measurements on the HfO_2_ device, both set voltage ranges (small range and large range) yield qualitatively very similar conductance curves. Here, $G/{G}_{\mathrm{max}}$ increases continuously with the number of SET pulses (${N}_{\mathrm{P}}=1{-}21$) until the LRS is reached and reduces continuously during the RESET process (${N}_{\mathrm{P}}=22{-}41$) after an initially large drop in the conductance during the first RESET pulse. The conductance curves of the two voltage modes of the HfO_2_ device differ mainly quantitatively, with a slightly larger nonlinearity and altered slope in the large-range measurement compared to the small-range measurement.

A similar conductance curve is observed in the small-range measurements on the HfO_1.8_ device (Fig. 7b) but with improved linearity and continuity, i.e., without a sudden drop in the conductance after the first RESET pulses. In the large voltage range, entirely different behavior of the conductance update is found. Here, an abrupt transition between the LRS ($G/{G}_{\mathrm{max}}\approx 1$) and HRS $\left(G/{G}_{\mathrm{max}}\approx 0\right)$ occurs after the first SET and RESET pulse, respectively, and in between, $G/{G}_{\mathrm{max}}$ changes slightly but almost linearly. This behavior of the conductance update curve differs qualitatively from the other three measurement sets, consistent with the current–voltage measurements in Fig. 2b.

## Discussion and conclusions

We presented trilayer HfO_x_/Al_2_O_3_/TiO_2_ memristive devices with two hafnium oxide stoichiometries (*x* = 1.8, *x* = 2) and analyzed their electrical characteristics for neuromorphic applications. We demonstrated the possibility of tuning the switching mechanism by altering the hafnium oxide stoichiometry and showed how the Al_2_O_3_ interlayer could extend the range in which area-type switching can be tuned. The Al_2_O_3_ interlayer leads to stable area-type switching at small voltages for both hafnium oxide stoichiometries. A change from an area-type switching mechanism to a filament-type is induced at a larger voltage amplitude in the HfO_1.8_ device, with strong indications for a transition from an n-type semiconductor to a p-type semiconductor. The origin of this transition and the switching mechanisms is discussed in detail and used for setting up a physics-based model capable of reproducing distinct features of the measured current–voltage characteristics. The model is based on an equivalent-circuit formulation and can be easily integrated into large-scale simulations for the design of memristive circuits. Quantitatively consistent with our model, we estimated an activation energy of $\approx 1.65 \; \mathrm{eV}\pm 0.05\;\mathrm{ eV}$ for Frenkel defects at the HfO_x_/Al_2_O_3_ interface. This is larger than the values of HfO_x_/TiO_x_ interfaces^47^ and can explain the stable area-type switching behavior we observe in the trilayer devices. The stabilization leads to various additional advantages in performance compared to previously presented HfO_x_/TiO_2_ bilayer devices^67^, such as the possibility of operating without current compliance and initial electroforming cycles in devices of both hafnium oxide stoichiometries.

Further, the state retention time and linearity in the conductance update were characterized. Depending on the stoichiometry and voltage range applied, we observed retention times from several days to months in the area-type devices and several years for the filamentary device with on–off ratios of 10^2^–10^3^ Consistent with the switching type, a binary conductance update is observed in the filamentary device, and an analog-type conductance update with many continuously accessible resistance states in the area-type devices. Compared to the other trilayer device configurations, the area-type HfO_1.8_ devices show much-improved linearity in the conductance update while keeping a large on–off ratio and a long retention time. These properties could make the devices promising for classical artificial neural networks such as deep neural networks and convolutional neural networks. Area-type switching in devices of this stoichiometry, and thereby, such a combination of properties, was essentially made possible by the stabilizing aluminum oxide layer. The nonlinear, time dependent resistance change in the HfO_2_ devices could be on the other hand suitable for implementing the STDP rule in spiking neural networks.

In conclusion, we demonstrated a technological methodology for tuning multilayer redox-based memristive devices. The insights gained on stabilizing the area-type switching mechanism are an essential step towards tailored devices unifying the beneficial characteristics of filamentary and area-type devices. We achieved promising properties with trilayer memristive elements and provided a model, which can be integrated into large-scale circuit simulations to support the design of memristive circuits for neuromorphic learning schemes in the future.

## Methods

### Device fabrication and setup

The TiN bottom electrode was deposited by reactive DC magnetron sputtering on top of a SiO_2_/Si substrate and afterward patterned with photolithography and a subsequent lift-off process. On top of the rear-side electrode, the titanium oxide (TiO_2_) layer was deposited using a reactive gas mixture of O_2_ and Ar with a volume ratio of 10/40. For the aluminum oxide layer (Al_2_O_3_), a 2-nm-thick Al layer was sputter deposited and subsequently thermally oxidized under an O_2_ atmosphere for 30 min. During the oxidization, an of Ar/O_2_ gas mixture was used with a volumetric ratio of 30/10 and a working pressure of 0.05 mbar. The stoichiometry of the polycrystalline monoclinic hafnium oxide (HfO_x_) layer was tuned to obtain either an atomic ratio of O/Hf of $x \approx 2$ deposited homogeneously on a rotating substrate or an atomic ratio of $x \approx 1.8$ with a static wedge deposition. An O_2_/Ar gas mixture with a volume ratio of 10/29 is used during the deposition process. The Au contact layer was electron-beam evaporated on the top of the functional trilayer and patterned via photolithography and a lift-off process. The devices were encapsulated by a 120-nm-thick SiO_2_ layer before the Al layer was evaporated and patterned to create the conduction lines. Details on the XPS measurements and the fabrication process are provided in Ref.^67^. The variation of electrical properties of reactively sputtered metal oxides has been investigated elsewhere^86^.

### Current–voltage characteristics

**Figure 8:**
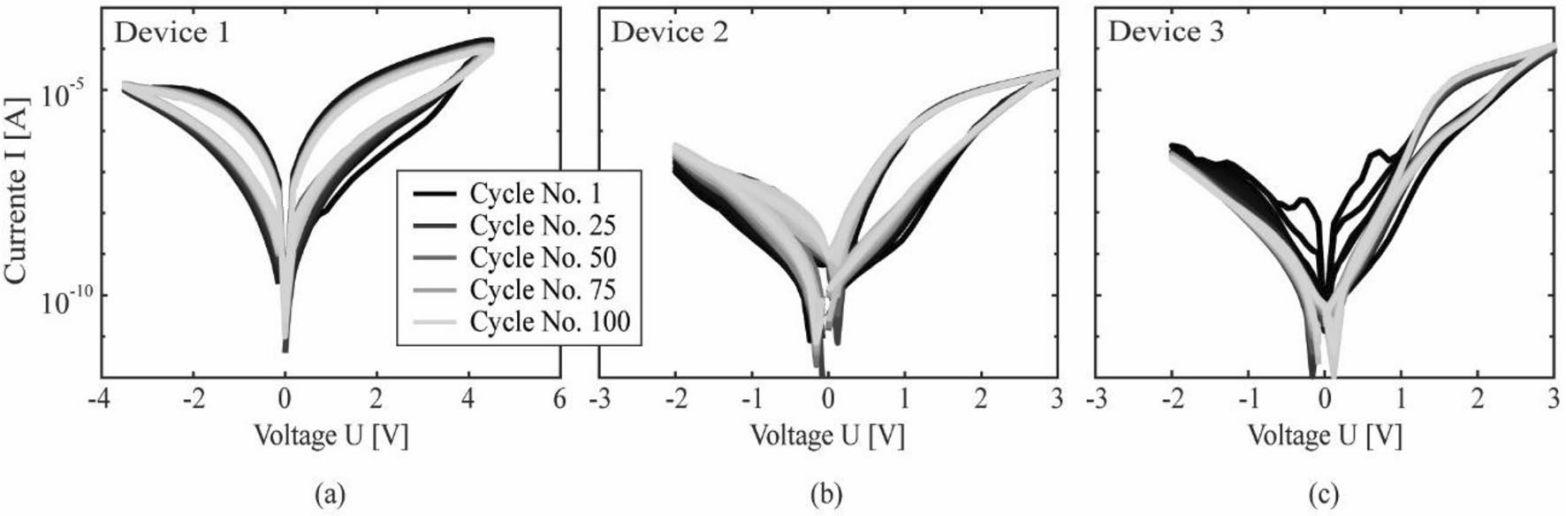
Current–voltage loops measured over a series of 100 periods on three representative example devices; (**a**) device 1: HfO_2_, device area 20^2^ µm^2^, large range, (**b**) device 2: HfO_2_, device area 25^2^ µm^2^, small range, and (**c**) device 3: HfO_1.8_, device area 10^2^ µm^2^, small range.

All electrical measurements were conducted with a source-measurement unit (Keysight B2901A). During the measurement, a voltage $U$ is applied to the top electrode and swept piecewise linearly between the start and end values ($U=0 \mathrm{V}$) and the respective maximum and minimum values. The maximum and minimum voltages are set to values just below the expected electrical breakdown voltage, which was determined in advance by measurements on similar devices. During the measurements, the rear-side electrode was grounded. The device-to-device variability was tested on several devices by probing the current over a series of 100 voltage cycles. Representative example measurements on three different devices are shown in Fig. 8.

### XRD measurements

X-ray diffraction (XRD) spectra were measured of two samples comprising different hafnium oxide layers on a Si substrate. The hafnium oxide layer was sputter deposited for 900 s on a rotating substrate to achieve a homogeneous HfO_2_ layer and in a static wedge-deposition mode to obtain a gradient HfO_x_ layer. From the two 4-in. test wafers, samples were selected from the same position in the center of the wafer, where a similar thickness of $\approx 30 \; \mathrm{nm}$ of hafnium oxide is expected and a composition of HfO_1.8_ of the wedged layer. The XRD system (Bruker D8 Discover, Bruker Corporation, USA) consists of a copper Kα radiation source and a Lynxeye XE-T detector with an energy resolution < 380 eV at 8 keV. The measured data are filtered to remove Kα-contributions, the background is subtracted, and they are normalized to the Si (100) peak at $2\theta =69^\circ$.

### Area dependency

For the analysis of the area dependency in Fig. 3, the resistance values were extracted from the I-U curves at a voltage of 1 V and only for the large-range measurements on the HfO_1.8_ device at 0.1 V. The measurements were performed on many devices and then averaged. An overview of the number of devices used for calculating the mean values and standard deviations is provided in Table 1 and the standard deviations are summarized in Table 2. Owing to the fabrication process, a significantly smaller number of devices with an HfO_1.8_ layer was available compared to the number of devices with an HfO_2_ layer.

**Table 1:** Overview of the number of devices used to calculate the mean values in Fig. 3 and the standard deviations in Table 2.

Device area *A* (${\mathrm{\mu m}}^{2}$)	HfO_2_		HfO_1.8_	
	Small range	Large range	Small range	Large range
${15}^{2}$	79	127	3	3
${20}^{2}$	108	145	4	5
${25}^{2}$	208	181	4	1
${35}^{2}$	316	179	4	6
${50}^{2}$	213	153	6	8

**Table 2:** Relative standard deviation of the resistance measurements in Fig. 3 in percent.

Device area *A* (${\mathrm{\mu m}}^{2}$)	HfO_2_				HfO_1.8_			
	Small range		Large range		Small range		Large range	
	LRS	HRS	LRS	HRS	LRS	HRS	LRS	HRS
${15}^{2}$	32	29	27	28	16	25	66	15
${20}^{2}$	35	36	16	14	20	14	27	6
${25}^{2}$	36	34	6	6	12	31	–	
${35}^{2}$	34	39	1	1	9	4	96	17
${50}^{2}$	36	36	5	5	10	6	60	19

### Pulse measurements

For all reading voltage pulses, amplitudes of 1.2 V (small range) and 0.1 V (large range) are used for the HfO_1.8_ device, and 1.2 V in both voltage ranges for the HfO_2_ device. For the linearity of conductance measurements (Fig. 7), SET/RESET voltage pulses with amplitudes of 2.5 V/− 1.5 V (small range) and 3.5 V/− 3 V (large range) are applied to the HfO_2_ device and 2.5 V/− 1.5 V (small range) and − 3 V/3 V (large range) to the HfO_1.8_ device. We used pulse lengths of $50 \; \mathrm{ms}$ with a frequency of 150 ms. For the retention test, we used amplitudes of the initial SET pulses of 3.5 V (small range) and 5 V (large range) for the HfO_2_ device and 3 V (small range) and − 2 V (large range) for the HfO_1.8_ device. The pulse lengths were 10 ms (small range) and 50 ms (large range) for the HfO_2_ device and 10 ms for the HfO_1.8_ device in both voltage ranges.

### Retention fits

The values of the parameters $\alpha$ and $\beta$, which result from the fit of the power function ${R}_{\mathrm{on}}/{R}_{\mathrm{off}}=\beta {t}^{\alpha }$ to the measured values of ${R}_{\mathrm{on}}/{R}_{\mathrm{off}}$ as a function of time *t* in Fig. 6 are provided in the following Table 3. For calculating the resistance ratios ${R}_{\mathrm{on}}/{R}_{\mathrm{off}}$, the on-state resistances ${R}_{\mathrm{on}}$ are normalized to the measured off-state resistances ${R}_{\mathrm{off}}=290 \;\;\mathrm{M\Omega }$ (HfO_2_, small range), ${R}_{\mathrm{off}}=0.7 \;\;\mathrm{M\Omega }$ (HfO_2_, large range) and ${R}_{\mathrm{off}}=80 \;\;\mathrm{M\Omega }$ (HfO_1.8_, small range), ${R}_{\mathrm{off}}=0.16 \;\;\mathrm{M\Omega }$ (HfO_1.8_, large range).

**Table 3:** Values of the fit parameters for the function ${R}_{\mathrm{on}}/{R}_{\mathrm{off}}=\beta {t}^{\alpha }$, plotted in Fig. 6.

	HfO_2_		HfO_1.8_	
Fit parameter	Small range	Large range	Small range	Large range
$\alpha$	$3.023\cdot {10}^{-5}$	$1.754\cdot {10}^{-3}$	$2.176\cdot {10}^{-3}$	$1.142\cdot {10}^{-2}$
$\beta$	$6.944\cdot {10}^{-1}$	$3.603\cdot {10}^{-1}$	$3.839\cdot {10}^{-1}$	$-6.427\cdot {10}^{-3}$

### Vacancy densities in area-type devices

The density of oxygen sites ${n}_{\mathrm{max}}={N}_{\mathrm{O}}/{V}_{\mathrm{c}}$ of ideal monoclinic HfO_2_ is estimated from the number ${N}_{\mathrm{O}}=4$ oxygen ions within one unit cell with volume ${V}_{\mathrm{c}}\approx (5 \;\mathrm{nm})^{2}$ to ${n}_{\mathrm{max}}=3.2\cdot {10}^{25} \;{\mathrm{m}}^{-3}$. Considering the fractions of < 1% and 10% of occupied oxygen sites from the stoichiometry measured via XPS, we estimate for HfO_2_ a density of stoichiometric vacancies of $\left[{V}_{\mathrm{O},\mathrm{s}}\right]<3.2\cdot {10}^{23} \;{\mathrm{m}}^{-3}$ and for HfO_1.8_ a density of $\left[{V}_{\mathrm{O},\mathrm{s}}\right]=3.2\cdot {10}^{24} \;{\mathrm{m}}^{-3}$. These vacancies can occupy various stable charged states, ${V}_{\mathrm{O}}^{0}$, ${V}_{\mathrm{O}}^{1+}$ and ${V}_{\mathrm{O}}^{2+}$, following the reactions ${V}_{\mathrm{O}}^{0}\to {V}_{\mathrm{O}}^{1+}+{e}^{-}$ and ${V}_{\mathrm{O}}^{1+}\to {V}_{\mathrm{O}}^{2+}+{e}^{-}$. The corresponding densities $[{V}_{\mathrm{O}}^{0}]$, $[{V}_{\mathrm{O}}^{1+}]$, $[{V}_{\mathrm{O}}^{2+}]$ can be calculated considering their ionization energies relative to the conduction band edge^65^. With activation energies of ${E}_{\mathrm{O}}^{0\to 1}\approx 0.63 \;\mathrm{eV}$ for the neutral-single charge transition^57^, and ${E}_{\mathrm{O}}^{1\to 2}=0.8 \;\mathrm{eV}$ for the single charge-double charge transition^57^, we obtain at $U = 0 \mathrm{V}$ concentrations of $[{V}_{\mathrm{O}}^{+2}]\approx \left[{V}_{\mathrm{O}}\right]$ for HfO_2_ and HfO_1.8_. For estimating the change of the electric-field induced vacancy density as a function of time, we consider the following rate equation^47^

1 $$\frac{\mathrm{d}[{V}_{\mathrm{O}}^{2+}]}{\mathrm{d}t}={v}_{0}{N}_{0,\mathrm{s}}\mathrm{exp}\left(-\frac{q{E}_{\mathrm{V}}-0.5zaqE}{{k}_{\mathrm{B}}T}\right),$$

with the phonon frequency ${v}_{0}$, the charge number $z=+2$ and hopping distance $a\approx 0.5 \;\mathrm{nm}$ of oxygen vacancies, the positive elementary charge $q$, the electric field $E$ across the HfO_x_ layer, the Boltzmann constant ${k}_{\mathrm{B}}$, the temperature $T=300 \; \mathrm{K}$, and the effective formation energy ${E}_{\mathrm{V}}$ of ${V}_{\mathrm{O}}^{2+}$. The electric field $E$ is obtained from the voltages solved for in the compact model. Because the reaction occurs at the HfO_x_/AlO_x_ interface and not in the entire volume, we use an effective volume density ${N}_{0,\mathrm{s}}={n}_{\mathrm{max}}\cdot {t}_{\mathrm{s}}/{t}_{\mathrm{HfO}} \approx 5\cdot {10}^{24}\;{\mathrm{m}}^{-3}$ of oxygen sites, scaling the ideal density of oxygen sites ${n}_{\mathrm{max}}$ by the ratio of surface thickness ${t}_{\mathrm{s}}$ to film thickness ${t}_{\mathrm{HfO}}.$ Further we assume that ${t}_{\mathrm{s}}\approx a$, the hopping distance of the vacancies. Details on the parameters are provided in “Simulation parameters”.

### Physics-based model

In the following, all equations used for the compact model are presented and explained. The contributions of the Al_2_O_3_ and the TiO_2_ layer to the current–voltage characteristics are considered by one effective resistance ${R}_{\mathrm{eff}}$ with a corresponding current ${I}_{\mathrm{eff}}$. This current depends on the voltage amplitude ${U}_{\mathrm{eff}}$ over these layers and is scaled by the fitting parameter ${I}_{\mathrm{eff},0}$^67^

2 $${I}_{\mathrm{eff}}:={I}_{\mathrm{eff},0}\mathrm{sinh}\left({U}_{\mathrm{eff}}\right).$$

Within the hafnium oxide layer, we consider the drift of mobile point defects and define a normalized concentration ${x}_{\mathrm{r}}$ ($0\le {x}_{\mathrm{r}}\le 1$) via the maximum concentration ${x}_{\mathrm{max}}$ and the minimum concentration ${x}_{\mathrm{min}}$ of mobile dopants at the Au/HfO_x_ interface

3 $${x}_{\mathrm{r}}:=\frac{x-{x}_{\mathrm{min}}}{{x}_{\mathrm{max}}-{x}_{\mathrm{min}}}\; \mathrm{with} \;{x}_{\mathrm{min}}\le x\le {x}_{\mathrm{max}}.$$

The average dopant concentration is then given by

4 $$\overline{x }:=\frac{1}{2}\left({x}_{\mathrm{max}}+{x}_{\mathrm{min}}\right).$$

The change of dopant concentration $x$ with time *t* at the Au/HfO_x_ interface is approximated using the (net) ionic current ${I}_{\mathrm{x}}$, the charge number $z$ of the drifting dopants, the cross-section area ${A}_{\mathrm{c}}$ of the conducting channel, and the channel length, which equals the thickness ${t}_{\mathrm{HfO}}$ of the HfO_x_ layer^87^

5 $$\frac{\partial x}{\partial t}:=\frac{{I}_{\mathrm{x}}}{zq{A}_{\mathrm{c}}{t}_{\mathrm{HfO}}}\; \mathrm{with}\; {I}_{\mathrm{x}}=zq{A}_{\mathrm{c}}\overline{x }\overline{v }.$$

For the area-type case, the cross-section area ${A}_{\mathrm{c}}$ is set to the active device area ${A}_{\mathrm{c}}=A$, while for filamentary conduction, it is set to the cross-section area ${A}_{\mathrm{f}}$ of the filament ${A}_{\mathrm{c}}={A}_{\mathrm{f}}=\pi {r}_{\mathrm{f}}^{2}$ with the filament radius ${r}_{\mathrm{f}}$. The drift velocity $\overline{v }$ has been formulated by Mott and Gurney^66^ as a function of the applied electric field *E* across the HfO_x_ layer

6 $$\overline{v }={v}_{0}a\mathrm{exp}\left(\frac{\Delta W}{{k}_{B}T}\right)2\mathrm{sinh}\left(\frac{zqaE}{2{k}_{B}T}\right), \;\;\mathrm{with } \;\; E=\frac{{U}_{\mathrm{x}}}{{t}_{\mathrm{HfO}}},$$

given by the voltage ${U}_{\mathrm{x}}$ across the HfO_x_ layer with a thickness ${t}_{\mathrm{HfO}}$. In this equation, ${v}_{0}$ is the attempt frequency (phonon frequency) for overcoming the potential barrier $\Delta W$, which separates the local minima. The distance between the local minima is given by the hopping distance $a$. The Boltzmann constant is denoted by ${k}_{\mathrm{B}}$ and the temperature by $T$. The electric resistance of the HfO_x_ layer is approximated to be independent of *x* for area-type conduction

7 $${R}_{\mathrm{x}}=\frac{{t}_{\mathrm{HfO}}}{\left|z\right|q{\mu }_{\mathrm{n}}A\overline{x} } ,$$

and linearly dependent on *x* for filamentary-type conduction

8 $${R}_{\mathrm{x}}\left(x\right)={R}^{\mathrm{LRS}}{x}_{\mathrm{r}}+{R}^{\mathrm{HRS}}\left(1-{x}_{\mathrm{r}}\right),$$

with the electric resistance ${R}^{\mathrm{LRS}}$ of the low resistance state, and ${R}^{\mathrm{HRS}}$ of the high resistance state

9 $${R}^{\mathrm{LRS}}=\frac{{t}_{\mathrm{HfO}}}{\left|z\right|q{\mu }_{\mathrm{p}}{A}_{\mathrm{f}}{x}_{\mathrm{max}}}, {R}^{\mathrm{HRS}}=\frac{{t}_{\mathrm{HfO}}}{\left|z\right|q{\mu }_{\mathrm{p}}{A}_{\mathrm{f}}{x}_{\mathrm{min}}}.$$

Here, the electron and hole mobilities are denoted as ${\mu }_{\mathrm{n}}$ and ${\mu }_{\mathrm{p}}$, respectively. For a phenomenological description of the current through the Schottky contact at the Au/HfO_x_ interface, a Schockley-like expression is used^88^

10 $${I}_{\mathrm{D}}=\left\{\begin{array}{c}{I}_{\mathrm{D},0}\left(\mathrm{exp}\left(\frac{q{U}_{\mathrm{D}}}{n\left(x\right){k}_{\mathrm{B}}T}\right)-1\right) \quad \mathrm{for}\quad U-{U}_{\mathrm{D}}\ge 0\\ -{I}_{\mathrm{D},0}\left(\mathrm{exp}\left(-\frac{{\alpha }_{\mathrm{D}}q{U}_{\mathrm{D}}}{{k}_{\mathrm{B}}T}\right)-1\right) \quad \mathrm{for}\quad U-{U}_{\mathrm{D}}<0\end{array}\right. ,$$

with the voltage ${U}_{\mathrm{D}}$ across the interface, the applied voltage $U$, the ideality factor $n\left(x\right)$, and a fitting factor ${\alpha }_{\mathrm{D}}$ included to describe the reversed current. The saturation current ${I}_{\mathrm{D},0}$ is described by^88^

11 $${I}_{\mathrm{D},0}={A}^{*}{A}_{\mathrm{c}}{T}^{2}\mathrm{exp}\left(-\frac{q{\varphi }_{\mathrm{D}}\left(x\right)}{{k}_{\mathrm{B}}T}\right),$$

with the effective Richardson constant ${A}^{*}=1.202\cdot {10}^{6} \mathrm{A}/(\mathrm{m}^{2}\mathrm{K}^{2})$. The effective Schottky barrier ${\varphi }_{\mathrm{D}}\left(x\right)$ and the ideality factor $n\left(x\right)$ are described as functions of the concentration $x$ of mobile dopants at the Au/HfO_x_ interface to cover various effects, which can cause a change in the barrier height. In linear approximation, it is

12 $$\begin{aligned} {\varphi }_{\mathrm{D}}\left(x\right) & ={\varphi }_{\mathrm{D}}^{\mathrm{HRS}}{x}_{\mathrm{r}}+{\varphi }_{\mathrm{D}}^{\mathrm{LRS}}\left(1-{x}_{\mathrm{r}}\right), \\ n\left(x\right) &={n}^{\mathrm{HRS}}{x}_{\mathrm{r}}+{n}^{\mathrm{LRS}}\left(1-{x}_{\mathrm{r}}\right). \end{aligned}$$

Here, ${\varphi }_{\mathrm{D}}^{\mathrm{HRS}}$ and ${\varphi }_{\mathrm{D}}^{\mathrm{LRS}}$ are the effective Schottky barriers in the high resistive state and low resistive state, respectively, and ${n}^{\mathrm{HRS}}$ and ${n}^{\mathrm{LRS}}$ the corresponding ideality factors. Considering Joule heating, we calculate the quasi-static equilibrium temperature *T* from

13 $$T={T}_{0}+\frac{UI}{{A}_{\mathrm{s}}{h}_{\mathrm{eff}}}:={T}_{0}+\frac{UI}{{R}_{\mathrm{T},\mathrm{eff}}},$$

with the environmental temperature ${T}_{0}=300\; \mathrm{K}$, the current $I={I}_{\mathrm{D}}$ through the device, the surface area ${A}_{\mathrm{s}}$ of the device through which heat is lost, and the effective heat-transfer coefficient ${h}_{\mathrm{eff}}$, which are unified in an effective thermal resistance ${R}_{\mathrm{T},\mathrm{eff}}$. Applying Kirchhoff’s circuit laws to the equivalent circuit in Fig. 5a, together with the above expressions, a system of equations is set up and solved numerically for the unknown voltages ${U}_{\mathrm{D}}$, ${U}_{\mathrm{x}}$, and ${U}_{\mathrm{eff}}$, as functions of the applied voltage.

### Simulation parameters

The parameters used for the simulations in Fig. 5 are summarized in Tables 4 and 5. The effective hopping distance $a\approx 0.5\;\mathrm{ nm}$ is estimated from the lattice constant^89^ and is similar to the length of the lowest energy hopping pathway of ${V}_{\mathrm{O}}^{2+}$^54^. For the filament radius ${r}_{\mathrm{f}}$, a value is used consistent with other studies on filamentary devices^47,65^. The value of the effective thermal resistance ${R}_{\mathrm{T},\mathrm{eff}}$ is adjusted to reach a maximum temperature of 550 K^63^ for the case of filamentary switching.

**Table 4:** Device-dependent fitting parameters used for the simulations in Fig. 5.

Parameter	HfO_1.8_		HfO_2_	
	Small range	Large range	Small range	Large range
${\phi }_{\mathrm{B}}^{\mathrm{HRS}} \left[\mathrm{eV}\right]$	0.65	0.31	0.73	0.51
${\phi }_{\mathrm{B}}^{\mathrm{LRS}} \left[\mathrm{eV}\right]$	0.58	0.34	0.63	0.41
${n}^{\mathrm{HRS}}$	12	7	12	24
${n}^{\mathrm{LRS}}$	11	3	9	15
${\alpha }_{\mathrm{D}}$	0.080	0.100	0.100	0.055
${I}_{\mathrm{el},\mathrm{O}} \left[\mathrm{A}/{\mathrm{m}}^{2}\right]$	$1\cdot {10}^{12}$	$2\cdot {10}^{14}$	$1\cdot {10}^{12}$	$1\cdot {10}^{12}$
$\Delta W [\mathrm{eV}]$	0.385	0.84	0.67	0.72
${x}_{\mathrm{min}} [1/{\mathrm{m}}^{3}]$	$5\cdot {10}^{23}$	$1\cdot {10}^{25}$	$1\cdot {10}^{18}$	$5\cdot {10}^{19}$
${x}_{\mathrm{max}} [1/{\mathrm{m}}^{3}]$	$6\cdot {10}^{24}$	$1.5\cdot {10}^{27}$	$1\cdot {10}^{19}$	$3\cdot {10}^{21}$

All parameters are defined in “Physics-based model”, where the model is described.

**Table 5:** Model parameters used for the simulations in Fig. 5.

Parameter	Value	Parameter	Value
${v}_{0} \left[\mathrm{Hz}\right]$	${10}^{-12}$^47^	${\mu }_{\mathrm{n}} \left[{\mathrm{m}}^{2}/(\mathrm{Vs})\right]$	${10}^{-5}$^67^
${t}_{\mathrm{HfO}} \left[\mathrm{nm}\right]$	3	${\mu }_{\mathrm{p}} \left[{\mathrm{m}}^{2}/(\mathrm{Vs})\right]$	${10}^{-4}$^64^
$a$[nm]	0.5	${r}_{\mathrm{f}} \left[\mathrm{nm}\right]$	50
${R}_{\mathrm{T},\mathrm{eff}}$[W/K]	$2.5\cdot {10}^{4}$	$A$[µm^2^]	35^2^
${T}_{0} \left[K\right]$	300	$z$	2

## Data Availability

The datasets used and/or analysed during the current study available from the corresponding author on reasonable request.
