# Author Correction: Multilayer redox-based HfO_x_/Al_2_O_3_/TiO_2_ memristive structures for neuromorphic computing

**DOI:** 10.1038/s41598-022-25502-w

**Published:** 2022-12-05

**Authors:** Seongae Park, Benjamin Spetzler, Tzvetan Ivanov, Martin Ziegler

**Affiliations:** Micro‑ and Nanoelectronic Systems, Institute of Micro and Nanotechnologies MacroNano, Technische Universität Ilmenau, Ilmenau, Germany

Correction to:* Scientific Reports* 10.1038/s41598-022-22907-5, published online 29 October 2022

The original version of this Article contained an error in the Funding section.

“Open Access funding enabled and organized by Projekt DEAL. The research is funded by the Carl-Zeiss Foundation via the Project MemWerk.”

now reads:

“Open Access funding enabled and organized by Projekt DEAL. The research is funded by the Carl-Zeiss Foundation via the Project MemWerk and the Deutsche Forschungsgemeinschaft (DFG, German Research Foundation) − Project-ID 434434223 − SFB 1461.”

The original Article has been corrected.

